# Bacteriome analysis of *Aggregatibacter actinomycetemcomitans*-JP2 genotype-associated Grade C periodontitis in Moroccan adolescents

**DOI:** 10.3389/froh.2023.1288499

**Published:** 2023-11-14

**Authors:** Vijaya Lakshmi Pavani Molli, Jamila Kissa, Divyashri Baraniya, Amina Gharibi, Tsute Chen, Nezar N. Al-Hebshi, Jasim M. Albandar

**Affiliations:** ^1^ Department of Periodontology and Oral Implantology, Maurice H. Kornberg School of Dentistry, Temple University, Philadelphia, PA, United States; ^2^ Department of Periodontology, Faculty of Dental Medicine, University of Hassan II, Casablanca, Morocco; ^3^ Oral Microbiome Research Laboratory, Maurice H. Kornberg School of Dentistry, Temple University, Philadelphia, PA, United States; ^4^ Department of Microbiology, Forsyth Institute, Cambridge, MA, United States

**Keywords:** biofilm, dysbiosis, high-throughput nucleotide sequencing, microbiota, periodontitis

## Abstract

**Background:**

Grade C (previously aggressive) periodontitis (GCP) in adolescents is prevalent in certain parts of Africa where it is associated with JP2 genotype, a highly virulent strain of *Aggregatibacter actinomycetemcomitans*. The aim of this study was to characterize the subgingival bacteriome in Moroccan subjects with GCP positive to *A. actinomycetemcomitans* JP2 genotype.

**Methods:**

Subgingival plaque samples were collected from shallow and deep pockets of 8 subjects with GCP (17.2 ± 1.5 years) and from gingival sulci of 13 controls with no periodontitis (14.6 ± 1.1 years). Identification and genotyping of *A. actinomycetemcomitans* was performed using PCR analysis of the *ltx* operon, while bacteriome profiling was done by 16S rRNA gene sequencing (V1–V3 region). Groups were compared in terms of microbial diversity, abundances, and dysbiosis.

**Results:**

The shallow and deep pocket sites from GCP cases had a significantly altered microbial composition compared to controls. Species associated with health included *Haemophilus parainfluenzae, Lautropia mirabilis, Streptococcus spp., Gemella spp.,* and *Rothia spp.* While known periodontal pathogens, including *Porphyromonas gingivalis*, *Tannerella forsythia*, *Treponema spp.* and *Fretibacterium spp.*, were significantly enriched in GCP, non-conventional taxa, including *Pseudomonas oral taxon C61* and *Enterobacter cloacae* were more abundant and showed stronger association with the disease. Less significant differences in abundances of individual taxa were observed between shallow and deep pockets. Overall dysbiosis measured in terms of Subgingival Microbial Dysbiosis Index (SMDI) differentiated between GCP and no-periodontitis with 95% accuracy.

**Conclusions:**

The results suggest that several periodontal pathogens involved in the adult-type periodontitis also play a role in JP2 genotype-associated GCP. The potential role of non-conventional taxa in the pathogenesis of GCP warrants further investigation.

## Introduction

Periodontitis encompasses a group of infectious diseases characterized by an inflammatory condition that leads to destruction of the periodontal supporting tissues, and eventual loss of the teeth (1). Grade C (previously aggressive) periodontitis (GCP) (2) is characterized by a disease onset at an early age, and presents as a localized forms that affects especially the molars and incisors, or a generalized form that affects most or all of the teeth (3). The etiology of this disease is not well understood. Present evidence indicates that microorganisms play a primary role in triggering a local inflammatory process which, in susceptible individuals, leads to significant and progressive periodontal tissue loss (4). The host immune response (5), and other factors (6) also play important roles in the development of this disease.

The subgingival bacterium *Aggregatibacter actinomycetemcomitans* has been widely implicated in the etiology of GCP (7). This microorganism is a fastidious, microaerophilic, gram-negative rod that possesses notable virulence factors, including a leukotoxin (LtxA*)* which has been shown to kill host leukocytes, induce degranulation in neutrophils and activate secretion of the pro-inflammatory cytokine IL-1β by macrophages (8–10). *A. actinomycetemcomitans* exhibits 2 different leukotoxic phenotypes: minimally and highly leukotoxic strains (11, 12). For instance, the *A. actinomycetemcomitans*-JP2 genotype is highly leukotoxic, which is attributed to a 530 bp deletion in the promotor region of the *ltx* gene operon (12). This genotype is found in subgingival samples of African subjects, and subjects with African ancestry, and is strongly associated with progressive loss of periodontal tissues in GCP (13–15). The genotype has recently been detected in non-African populations as well (16, 17).

Technological advances, including the advent of next generation sequencing technologies, have revolutionized our understanding of the oral bacterial communities (bacteriome) and their role in health and disease (18). Current understanding indicates that in periodontitis there is an imbalance in the composition and function of the subgingival bacteriome (dysbiosis), rather than an infection with specific pathogens (19). This premise seems pertinent also for GCP, where the disease, especially its generalized form, has been associated with a complex subgingival microbial signature that resembles that found in adult periodontitis (7, 20–23).

Our understanding of the bacteriome of GCP is still lacking. Particularly, little is known about the bacteriome associated with JP2 genotype-associated disease. A previous pilot study involving 4 Moroccan GCP patients found that 2 of these were JP2-genotype positive, and reported that the JP2 genotype represented only a minor component of a complex subgingival microbiota (24). Longitudinal studies show that carriage of JP2 genotype is a risk factor of onset and progression of periodontitis (13); suggesting that it plays a role in early stages of the disease as a keystone pathogen, but is then overgrown by a dysbiotic microbiome. Therefore, a more detailed knowledge of the microbial environment associated with the JP2-genotype infection seems pertinent to a better understanding of the pathogenesis of this disease.

The aim of this study was to characterize the subgingival bacteriome in shallow and deep pockets in GCP subjects as compared to subjects without periodontitis (controls) with particular emphasis on subjects positive to *A. actinomycetemcomitans*-JP2 genotype.

## Materials and methods

This study was reviewed and approved by the Institutional Review Board (IRB) at Temple University (project #23242) and by the IRB at the University of Hassan II, Casablanca, Morocco. The children and their parents were provided with sufficient information about the study objectives and methods, and a signed consent form confirming voluntary participation in the study was obtained from the subjects or a legally authorized guardian.

### Study population

The study subjects were identified in a population survey, designed to study the dental health of school children in Morocco (3). In the survey, middle- and high-schools from different regions of Morocco were selected using a probability sampling design. As part of this survey, 984 adolescents 14–18 years old attending schools in the Moroccan cities of Tiznit, Dakhla, and Tangier were interviewed and underwent a comprehensive periodontal examination during the period 2016–1017. The examination consisted of measurement of the probing depth (PD), gingival recession, and bleeding on probing at six sites per tooth on all fully erupted permanent teeth, excluding third molars. The clinical attachment loss (CAL) was then calculated and used to identify subjects with GCP using established criteria (2, 3).

The criteria of inclusion of cases in this study were subjects diagnosed with GCP, tested positive for the *A. actinomycetemcomitans* JP2 genotype, and who have not had periodontal treatment or used systemic antibiotic during the previous 3 months. In this population, 45 subjects were diagnosed with GCP, and 8 of these fulfilled these criteria and were included in the study. In addition, 13 subjects without periodontitis (controls) were selected randomly from the same population. The clinical characteristics of the two groups are presented in Table 1.

**Table 1:** The clinical characteristics of the study subjects.

	Cases (*n* = 8)			Controls (*n* = 13)		
Females, no. (%)	5 (62%)			5 (38.5%)		
	Mean	S.D.	Range	Mean	S.D.	Range
Age	17.2	1.5	14–18	14.6	1.1	14–17
#Teeth with BOP	21.7	8.8	9–28	5.7	5.7	0–14
#Teeth with PD ≥ 4 mm	11.6	7.1	3–20	0.4	1.6	0–6
#Teeth with PD ≥ 5 mm	7.6	3.7	3–13	0.1	0.5	0–2
#Teeth with PD ≥ 6 mm	3.4	2.9	0–9	0.0	0.0	0–0
Maximum PD	8.4	2.4	5–12	3.1	0.5	3–5
Mean PD	3.8	0.7	2.7–4.5	2.2	0.3	1.8–3.0
#Teeth with LOA ≥ 4 mm	7.7	3.6	3–13	0.1	0.5	0–2
#Teeth with LOA ≥ 5 mm	3.7	2.8	0–9	0.0	0.0	0–0
#Teeth with LOA ≥ 6 mm	2.3	2.0	0–5	0.0	0.0	0–0
Maximum LOA	8.0	2.6	4–12	2.4	0.6	2–4
Mean LOA	3.2	0.4	2.7–3.5	1.9	0.2	1.7–2.2

BOP, bleeding on probing; LOA, loss of attachment; PD, probing depth.

### Subgingival plaque sample collection

In the GCP group, subgingival plaque samples were collected from 2 sites with shallow pockets (PD ≤ 4 mm) and 2 sites with deep pockets (PD ≥ 6 mm). In the control group, plaque samples were collected from two sites with PD ≤ 4 mm. The sites were isolated with cotton rolls, and any supragingival plaque was removed; then a sterile paper point was inserted into the depth of the pocket and left in place for 30 s. The paper points from each subject were pooled, separately for shallow and deep pockets in the cases, placed in 1 ml Tris EDTA buffer, and maintained at −20°C until analyzed.

### DNA extraction

The samples were vortexed vigorously and pelleted by centrifugation at 13,000 rpm for 10 min. The pellets were each re-suspended in 162 μl PBS with 18 μl Metapolyzme (Sigma, USA), and incubated at 35°C for 4 h. DNA was then extracted using the Invitrogen Purelink Genomic DNA extraction kit (Thermo Fisher Scientific, USA) according to manufacturer’s instructions, quantified using a Qubit 2 Fluorometer (Thermo Fisher Scientific, USA) and stored at −80°C.

### Identification and genotyping of *A. actinomycetemcomitans*

*A. actinomycetemcomitans* was identified and genotyped by PCR analysis of the *ltx* operon using the JP2-F3 (5’—TCTATGAAT GGAAACTTGTTCAGA AT−3’) and JP2-R2 (5’—GAA TAA GAT AAC CAA ACC ACA ATA TCC—3’) primers described by Youshida et al (25). While these primers had been originally reported to selectively detect JP2 genotype strains by amplifying a 163-bp fragment, we found they also amplify a 691 bp fragment in non-JP2 genotype strains, which was confirmed by *in silico* analysis. PCR reactions were performed in Platinum™ II Hot-Start Green PCR Master Mix (Thermo Fisher Scientific, USA), using the following thermal cycling parameters: initial denaturation at 94°C for 2 min, followed by 45 cycles of denaturation at 94°C for 15 s, annealing at 50°C for 15 s, and extension at 68°C for 1 min. Fragment analysis was performed using standard agarose electrophoresis. DNA extracted from *A. actinomycetemcomitans* non-JP2 strain NCTC 9710 (Public Health England) and JP2 strain ATCC 700685 (American Type Culture Collection, USA) was used as positive control.

### 16S rRNA sequencing and bioinformatic analysis

Library preparation and sequencing were done at the Australian Centre for Ecogenomics, as described previously (26). Briefly, the degenerate primers 27FYM (27) and 519R (28) were used to prepare indexed libraries of the V1–V3 region of the 16S rRNA gene that were subsequently sequenced on an Illumina Miseq platform using 2*300 bp chemistry.

Paired reads were merged with PEAR (29) and further processed, including quality-filtration, alignment and chimera check, using mothur software package (30) as previously described. The high-quality, non-chimeric sequences were classified to the species-level employing our BLASTN-based algorithm (31). Species represented by less than 100 reads across samples were filtered out. QIIME (Quantitative Insights Into Microbial Ecology) (32) was used for generation of taxonomy plots and calculation of species richness and alpha diversity. Taxonomic read counts were centered log-ratio (CLR) transformed and used for downstream analyses: (1) beta diversity analysis, namely principal component analysis (PCA) and Permutational Multivariate Analysis of Variance (PERMANOVA) using the R packages Vegan, Phyloseq (33) and Microbiome (34); and (2) differential abundance analysis using Microbiome Multivariable Associations with Linear Models (MaAsLin2) (35). Subgingival Microbial Dysbiosis Index (SMDI) as a summary statistic of microbial profiles and measure of dysbiosis was calculated from CLR transformed data as previously described (36). Selected significant results were plotted using ggplot2 package in R.

## Results

### 16S sequencing data pre-processing statistics

A total of 1,588,128 paired sequences were obtained (49,629 ± 31472 reads per sample). On average, 88.32% of reads were successfully merged with PEAR and ∼45% were retained after quality filtration and chimera removal steps. Around 90% of the high-quality merged reads were successfully classified to species level. Detailed statistics for preprocessing of each sample is provided in Supplementary File S1. Raw sequences were submitted to Sequence Read Archive (SRA) under project # PRJNA1028864.

### *A. actinomycetemcomitans* infection and genotype status

In the GCP group, 5 subjects (62.5%) were positive for the JP2 genotype but negative for the non-JP2 genotype, while 3 subjects (37.5%) were positive for both the JP2 and non-JP2 genotypes. In the control group, 1 subject (7.7%) was positive for both genotypes; 5 subjects (38.5%) were negative for both strains; 5 subjects (38.5%) were positive for the non-JP2 genotype and negative for the JP2 genotype; and 2 subjects (15.4%) were JP2 genotype positive and non-JP2 genotype negative.

### Distinct average subgingival bacteriome profile identified in GCP

**Figure 1:**
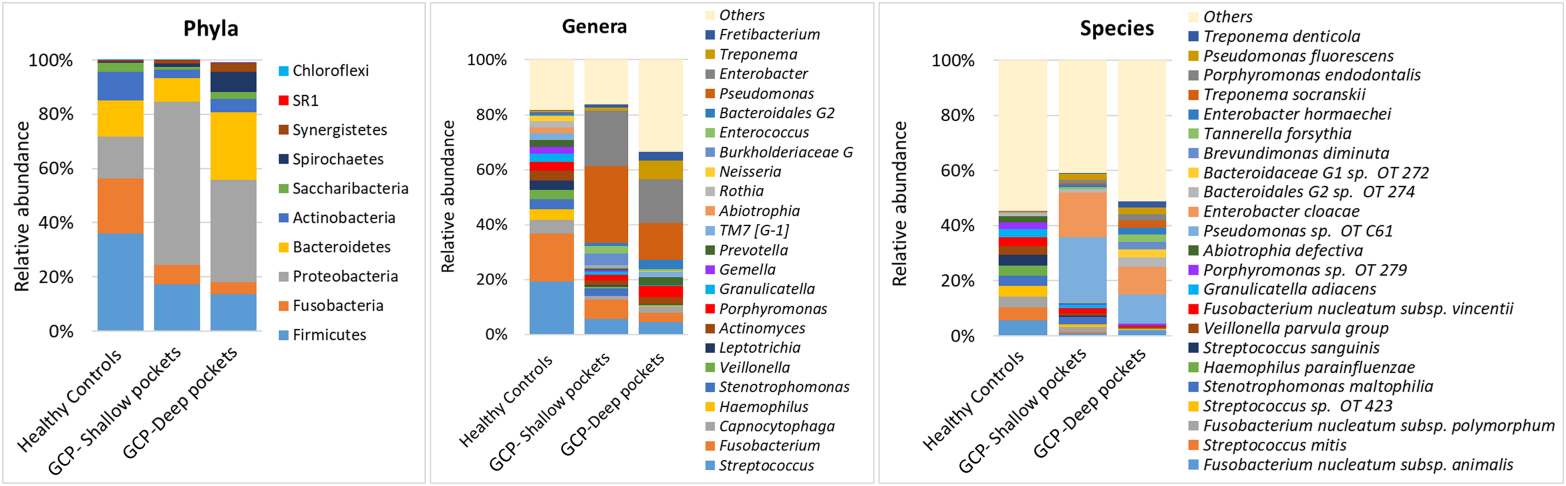
Average microbial profiles. DNA extracted from subgingival plaque samples was sequenced for the V1–V3 region of the 16S rRNA gene using 2 × 300 bp chemistry. The resultant sequences were merged, quality-filtered and classified to the species level using a BLASTn-based algorithm. The stacked bars show the average relative abundances of all phyla, top genera and top species identified in the study groups.

A total of 10 bacterial phyla, 109 genera and 308 species were identified in the samples, with a mean of 8.79 ± 1.13 phyla, 60.32 ± 12.96 genera and 152.32 ± 35.13 species per pooled sample*.* The relative abundances and detection frequencies of the identified phyla, genera and species per sample and per group are provided in Supplementary Files S2–S4. The average relative taxonomic profiles of each of the study groups are presented in Figure 1. While Proteobacteria, Firmicutes*,* Bacteroidetes and Fusobacteria were overall the most abundant phyla, accounting for >80% of the average bacteriome, their relative abundances substantially varied between the groups with Proteobacteria being the most abundant in GCP (followed by Bacteroidetes and Firmicutes in the deep and shallow pockets, respectively), and Firmicutes followed by Fusobacteria in the controls.

Differences were more evident at the genus and species levels. *Streptococcus* was the most abundant genus in the controls, followed by *Fusobacterium*, *Capnocytophaga, Haemophilus, Stenotrophomonas, Veillonella,* and *Leptotrichia,* together constituting on average 56% of the microflora. In the GCP group, *Enterobacter* and *Pseudomonas* were the most abundant genera accounting alone for 48% and 29% of the average bacteriome in the shallow and deep pockets, respectively, followed by *Treponema*, *Streptococcus*, *Porphyromonas*, *Fusobacterium*, *Bacteroidales* genus 2, and *Fretibacterium.* At the species level, *Fusobacterium nucleatum* was the most abundant in the controls, followed by *S. mitis*, *Streptococcus* oral taxon 423, *Stenotrophomonas maltophilia, Heamophilus parainfluenzae, Streptococcus sanguinis, Veilonella parvula group,* and *Granulicatella adiacens*. In GCP, the most abundant species were *Pseudomonas oral taxon C61* and *Enterobacter cloacae*, constituting together about 40% and 20% on average in the shallow and deep pockets, respectively, followed by *Bacteroidales* oral taxa 274 & 272, *Brevundimonas diminuta*, *Tannerella forsythia*, *Enterobacter hormaechei*, *Treponema socranskii*, *Porphyromonas endodontalis*, *Pseudomonas fluorescens* and *Treponema denticola*.

### GCP associated with altered microbial diversity but not dispersion

**Figure 2:**
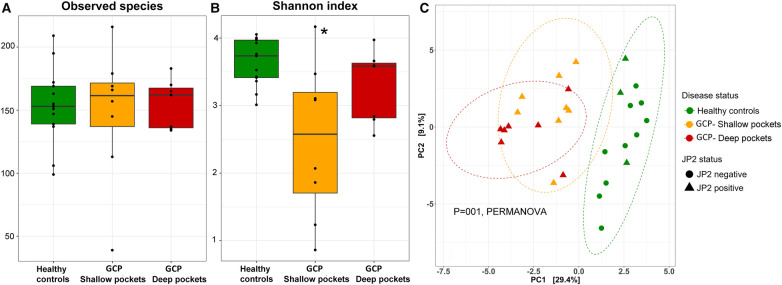
Species richness and diversity. Species count table were rarified and used to calculate observed species richness (**A**), and alpha diversity [Shannon index, (**B**)]. The data were then centered log-ratio transformed and used for beta-diversity analysis based on Principle Component Analysis [PCA plot, (**C**)]. Significance of differences between the groups for (**A**) and (**B**) were sought using pairwise Mann-Whitney or Wilcoxon signed rank test as appropriate; *P ≤ 0.05. Significance of differences between the clusters in (**C**) were assessed with PERMANOVA.

There were no differences between the groups in terms of species richness (Figure 2A), but both the shallow and deep pockets showed reduced alpha diversity (Shannon index) compared to the controls, although the difference was only statistically significant for the shallow pockets (Figure 2B). Beta-diversity analysis (PCA and PERMANOVA) revealed significant differences among the groups (Figure 2C), with the controls forming a separate cluster from the shallow and deep pockets (*P* = 0.001 for both contrasts). There was some separation between the shallow and deep pockets, but the difference did not reach statistical significance (*P* = 0.09). On the other hand, applying Permutational Multivariate Analysis of Dispersion (PERMDISP) to data identified no significant differences between the groups.

### Classical periodontal pathogens and non-conventional taxa enriched in GCP

**Figure 3:**
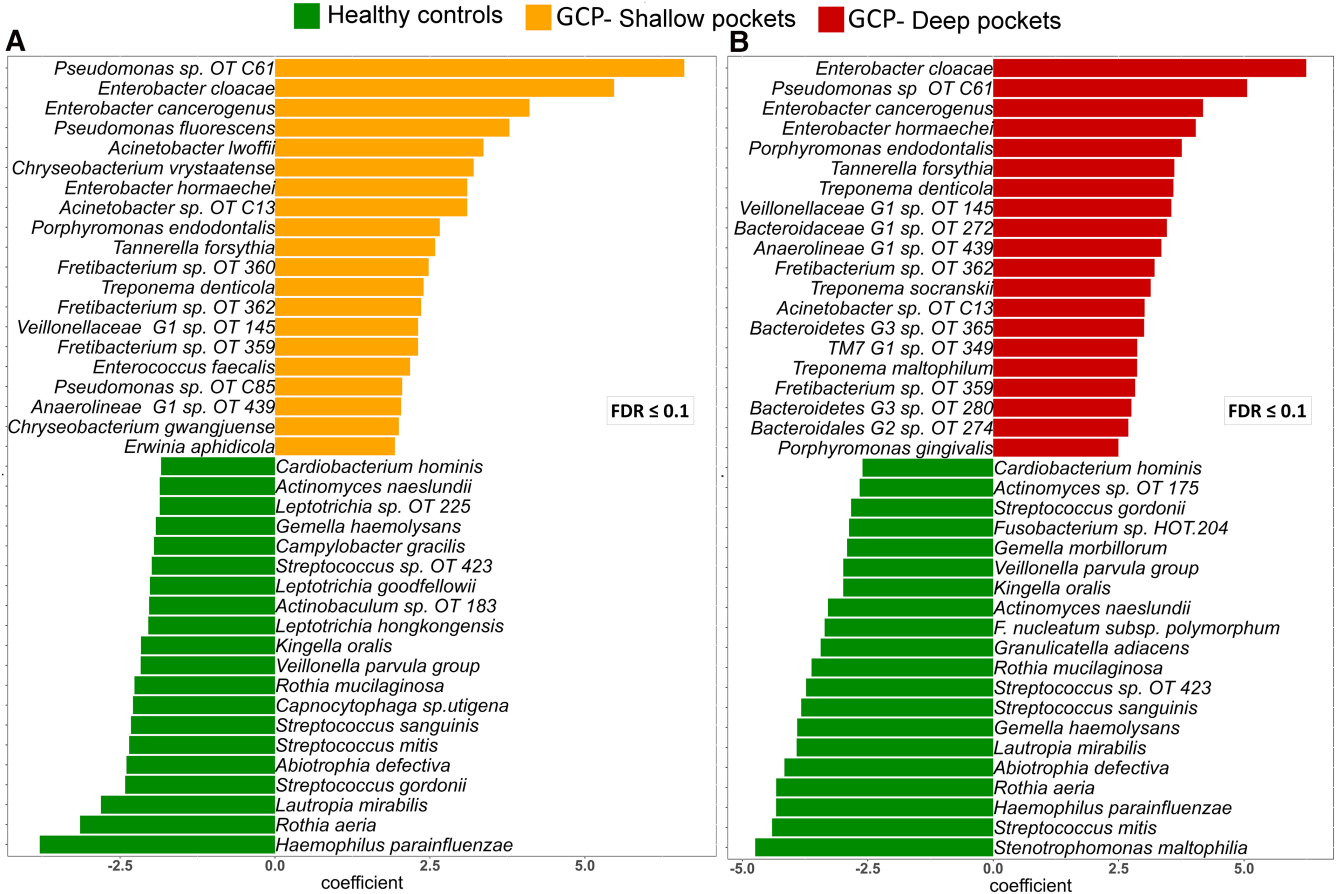
Top differentially abundant species between GCP and health. Centered log ratio (CLR) transformed data were analyzed with MaAsLin2 to identify differentially abundant species between the controls and shallow pockets from the GCP cases (**A**) and between the controls and deep pockets from the GCP cases (**B**) Differences with FDR ≤ 0.1 were considered significant. The top 40 species based on magnitude of association (coefficient) are shown in (**A**) and (**B**).

**Figure 4:**
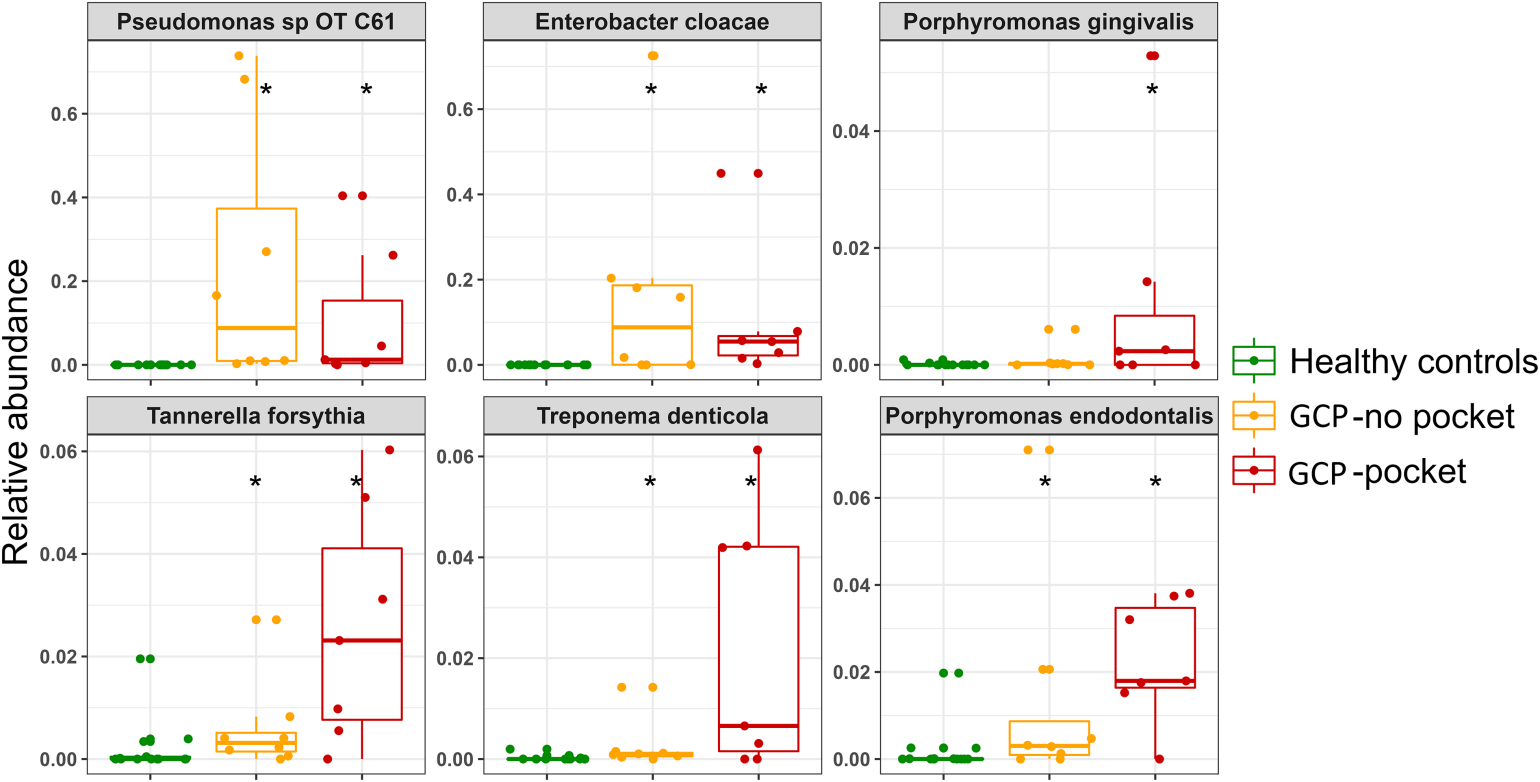
Relative abundances of selected species. Combined box and strip plots depicting the relative abundances of selected known periodontal pathogens as well as non-conventional species that were identified as significantly different between GCP cases and control subjects. *Statistically significant difference based on MaAsLin2 analysis; FDR ≤ 0.1.

Analysis with MaAsLin2 identified 46 and 86 differentially abundant genera and species, respectively, between the controls and shallow pockets, and 49 and 127 differentially abundant genera and species, respectively, between the controls and deep pockets at a false discovery rate (FDR) of 0.1 (Supplementary Files S5, S6). The top 20 differentially abundant genera in each contrast are presented in Supplementary Figure S1, and the corresponding top 40 differentially abundant species in each comparison are shown in Figure 3. Compared to both GCP subgroups, the controls had significantly higher abundance of known health-associated bacterial species including *H. parainfluenzae*, *S. mitis*, *Lautrophia mirabilis*, *S. sanguinis, V. parvula group, Rothia mucilaginosa*, *Rothia aeria*, *Kingella oralis*, *Streptococcus gordonii* and *Gemella haemolysans*. In contrast, known or putative periodontal pathogens were enriched in both the shallow and deep pockets of GCP compared to the controls including *T. forsythia*, *Treponema socranskii*, *P. endodontalis*, *T. denticola*, *P. gingivalis* and *Fretibacterium* spp. However, several non-conventional species were also abundant, and exclusively found, in the GCP samples including *Pseudomonas oral taxon C61*, *E. Cloacae*, *Enterobacter cancerogenus* and *Acinetobacter* oral taxon C13. Relative abundances of selected species identified as significantly associated with GCP are depicted in Figure 4.

No differentially abundant taxa between the shallow and deep pockets were found at an FDR ≤ 0.1. However, using a less stringent cutoff (FDR of 0.25 and *P*-value of 0.01, for genus and species-level comparisons, respectively) identified a few differences, mainly genus *Treponema* in association with the deep pockets and genera *Gemella*, *Rothia*, and *Ganulicatella* in addition to *S. mitis* and the non-conventional species *Stenotrophomonas maltophilia* in association with the shallow pockets (Supplementary Figure S2).

### SMDI discriminates between GCP and health with high accuracy

**Figure 5:**
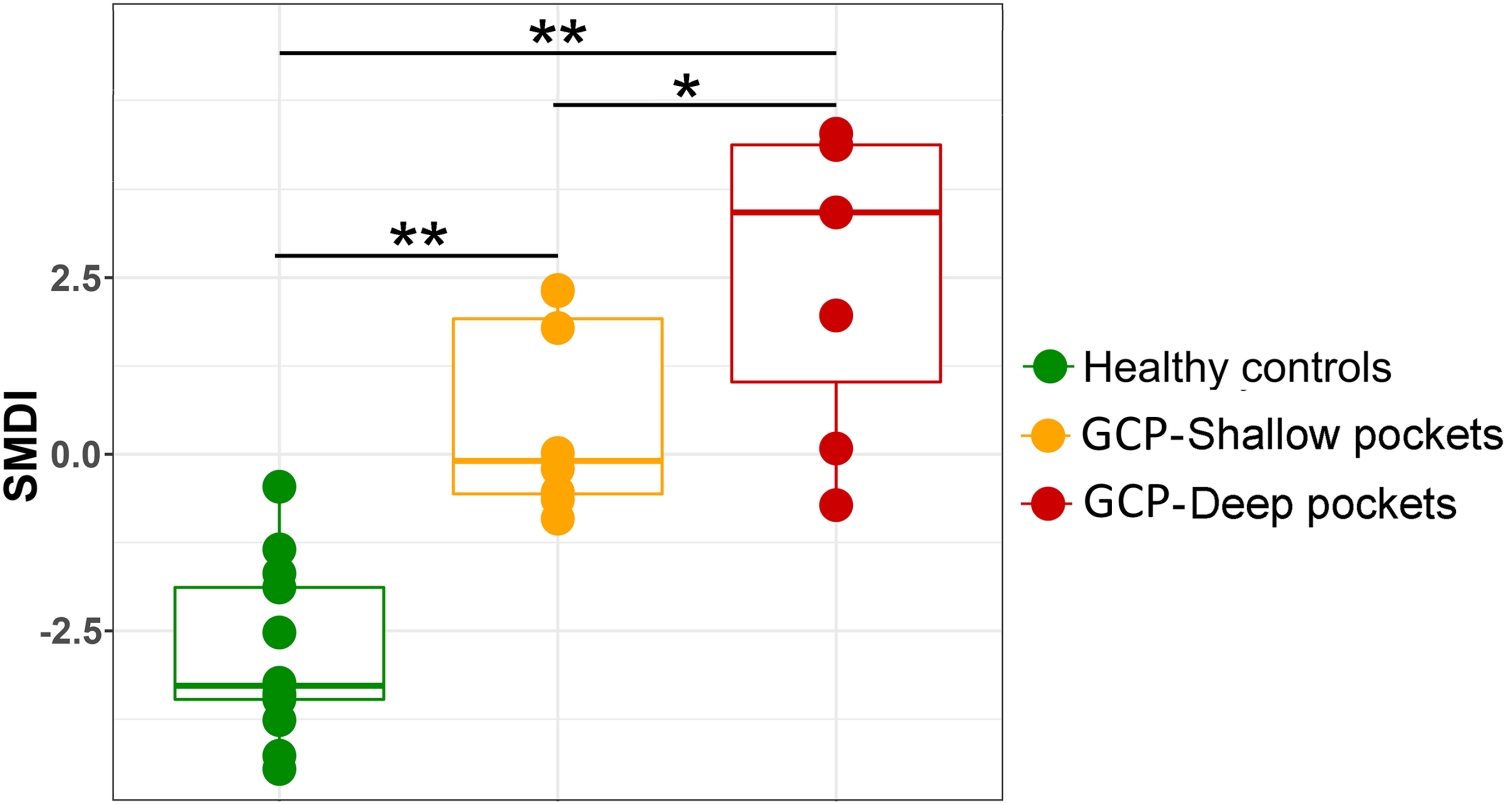
Subgingival microbial dysbiosis in GCP compared to health. Centered log ratio (CLR) transformed data were used to calculate SMDI for each sample as previously described (36) and presented as combined box and strip plot for each group. Significance of differences between the groups were sought using pairwise Mann-Whitney or Wilcoxon signed rank test as appropriate; *P ≤ 0.05; **P ≤ 0.01.

The level of subgingival microbial dysbiosis, measured in terms of our recently developed SMDI, differed significantly in pairwise comparison between the three groups, being highest in the deep pocket and lowest in the controls (Figure 5). Using an empirical SMDI value of 0 as diagnostic cutoff, discriminated between healthy sites and deep pockets with an accuracy of 95% and between healthy sites and shallow pockets with an accuracy of 81%. Using deep pocket data only, GCP cases were diagnosed with sensitivity of 85.7% and a specificity of 100% (95% accuracy).

## Discussion

Several studies have been conducted to explore the bacteriome of GCP (7, 20–23). However, to the best of our knowledge, the current study is the first attempt to focus on characterizing the bacteriome associated with JP2 genotype-associated GCP. This study included analysis of subgingival plaque samples collected from shallow and deep pockets of 8, previously untreated JP2 genotype-positive GCP cases and from gingival crevices of 13 controls using 16S rRNA gene sequencing. By applying a robust bioinformatic analysis approach to the data, we identified profound and important differences in the microbial community composition between the shallow and deep pockets in the GCP subjects, and between the GCP group and the controls.

Beta-diversity analysis (PCA plot) revealed complete separation between the GCP and controls, which has been previously demonstrated (20, 23). However, there were no differences in beta-dispersion between the 2 groups; i.e., the variation in bacteriome composition among the GCP cases was not different from that among the controls. Therefore, the GCP-associated bacteriome in this study did not follow the Anna Karenina principle (37), which is inconsistent with the findings in a recent study by Altabtbaei et al (20). Whether or not this is specific to JP2 genotype-associated GCP bacteriome entails further investigation. Interestingly, 3 subjects in the control group tested positive for the JP2 genotype, but still clustered with JP2-genotype negative samples. One possible explanation is that these subjects are yet to develop GCP since the average age of the controls was somewhat lower than that of the GCP cases. Alternatively, it may be that bacteriome dysbiosis is a prerequisite for JP2 genotype activity. This is an interesting hypothesis that warrants further exploration.

Previous studies have shown that GCP, particularly the generalized form, is associated with a dysbiotic subgingival bacteriome that shares similarities with that found in association with chronic periodontitis. Consistently, we found that several established and emerging periodontal pathogens are significantly more abundant in both shallow and deep probing sites in GCP compared to controls, including members of the “red complex” (*P. gingivalis*, *T. forsythia*, and *T. denticola*), *P. endodontalis*, *Fretibacterium* spp. and TM7 spp. Furthermore, applying our recently reported subgingival microbial dysbiosis index (SMDI) to the present data successfully discriminated between the GCP cases and controls with high accuracy (95%). SMDI was developed using a machine learning approach applied to data sets from studies on the bacteriome of chronic periodontitis, providing a diagnostic accuracy of 92%–96%. This striking similarity in the diagnostic accuracy of SMDI between chronic periodontitis and JP2 genotype-associated GCP, suggests that the two diseases share the features of subgingival microbial dysbiosis.

It may be hypothesized that GCP is a consequence of some kind of interactions between the JP2 genotype and subgingival bacteriome dysbiosis. One conceivable mechanism is that the JP2 genotype act as a keystone pathogen that subverts the immune response by its leukotoxicity effect that could disrupts microbial hemostasis, that in turn orchestrates a destructive immune response, consistent with the polymicrobial synergy and dysbiosis hypothesis (38). Indeed, in this study *A. actinomycetemcomitans* had a very low abundance in the subgingival plaque, which is an important feature of keystone pathogens. Another possibility is that the activity of the JP2 genotype is modulated by the bacteriome: in the presence of microbial dysbiosis, the JP2 genotype becomes more virulent, resulting in rapidly progressive form of periodontitis.

A novel finding in this study is the detection of non-oral gram negative facultatively anaerobic rods, including *Pseudomonas*, *Enterobacter* and *Acinetobacter* spp. in both shallow and deep pockets of the GCP cases. *Pseudomonas* oral taxon C61 and *E. Cloacae* together accounted for an average of 30% of the subgingival bacteriome of the GCP subjects. Species belonging to these same genera have been previously isolated from pockets of both chronic and GCP but not in such high abundance as in this material (39–41). It is not likely that detecting these species in the samples was due to, for example contamination during collection or processing of the samples, because these species were not detected in the control samples. However, since this study design is cross-sectional, it is not possible to infer on the role of these species in the pathogenesis of GCP in the present subjects. *Pseudomonas* oral taxon C61 is closely related to *Pseudomonas fluorescens* which, as well as *E. Cloacae,* is a ubiquitous species found in the environment and as a commensal organism in humans, however, in immunocompromised patients, they result in various opportunistic infections (42, 43). It is also notable that enteric rods have been shown previously to correlate with the level of *A. actinomycetemcomitans* and the severity of periodontal disease (40, 41). Therefore, it is possible that they play an opportunistic role in JP2 genotype-associated GCP.

In conclusion, in JP2 genotype-associated GCP, we identified a dysbiotic subgingival bacteriome that is somewhat similar to the bacteriome associated with chronic periodontitis. SMDI, which was originally developed for chronic periodontitis, characterized GCP cases with high accuracy. The interplay between subgingival dysbiosis and JP2 genotype infection, as well as the potential role of non-oral gram-negative rods in the pathogenesis of GCP warrants further investigation.

## Data Availability

The datasets presented in this study can be found in online repositories. The names of the repository/repositories and accession number(s) can be found below: https://www.ncbi.nlm.nih.gov/, PRJNA1028864.
