# Supplementary material for: Bacteriome analysis of Aggregatibacter actinomycetemcomitans-JP2 genotype-associated Grade C periodontitis in Moroccan adolescents
Source: Front Oral Health. 2023 Nov 14;4:1288499. doi: 10.3389/froh.2023.1288499 (PMC10682098; doi:10.3389/froh.2023.1288499)
Supplement: Supplementary file 7 [file Presentation1.pptx]

## Slide 1
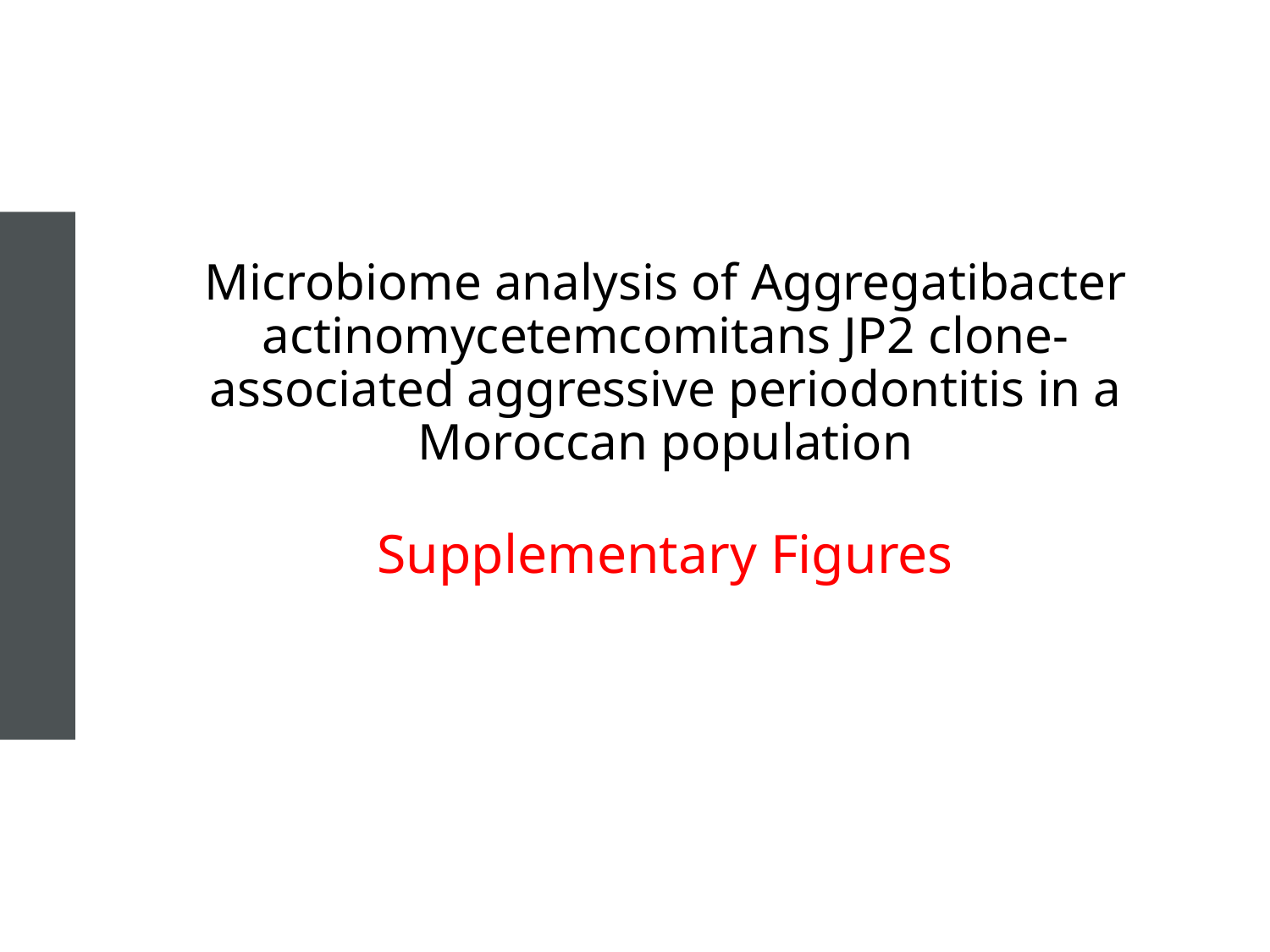

# Microbiome analysis of Aggregatibacter actinomycetemcomitans JP2 clone-associated aggressive periodontitis in a Moroccan population Supplementary Figures

## Slide 2
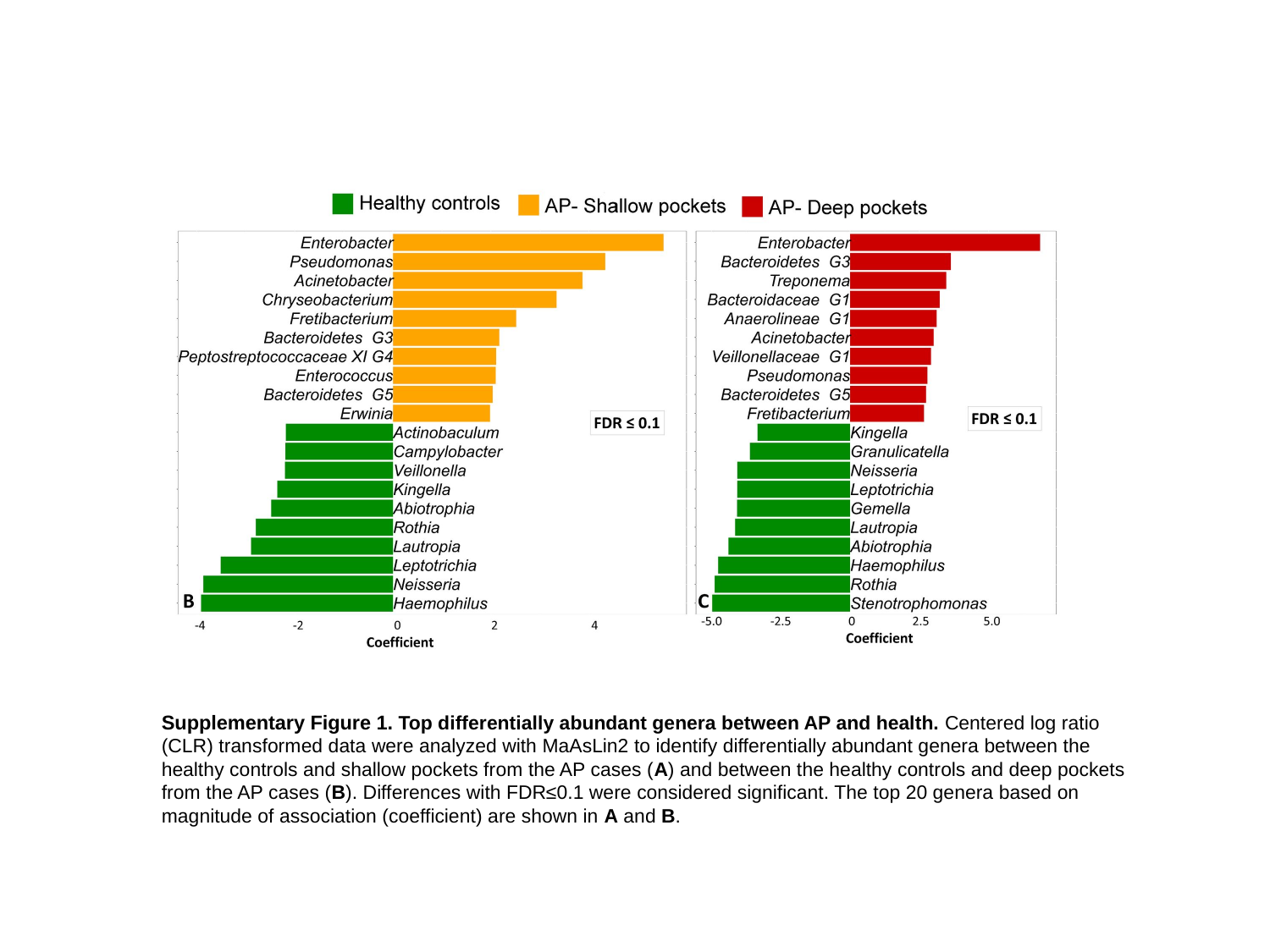

Supplementary Figure 1. Top differentially abundant genera between AP and health. Centered log ratio (CLR) transformed data were analyzed with MaAsLin2 to identify differentially abundant genera between the healthy controls and shallow pockets from the AP cases (A) and between the healthy controls and deep pockets from the AP cases (B). Differences with FDR≤0.1 were considered significant. The top 20 genera based on magnitude of association (coefficient) are shown in A and B.

## Slide 3
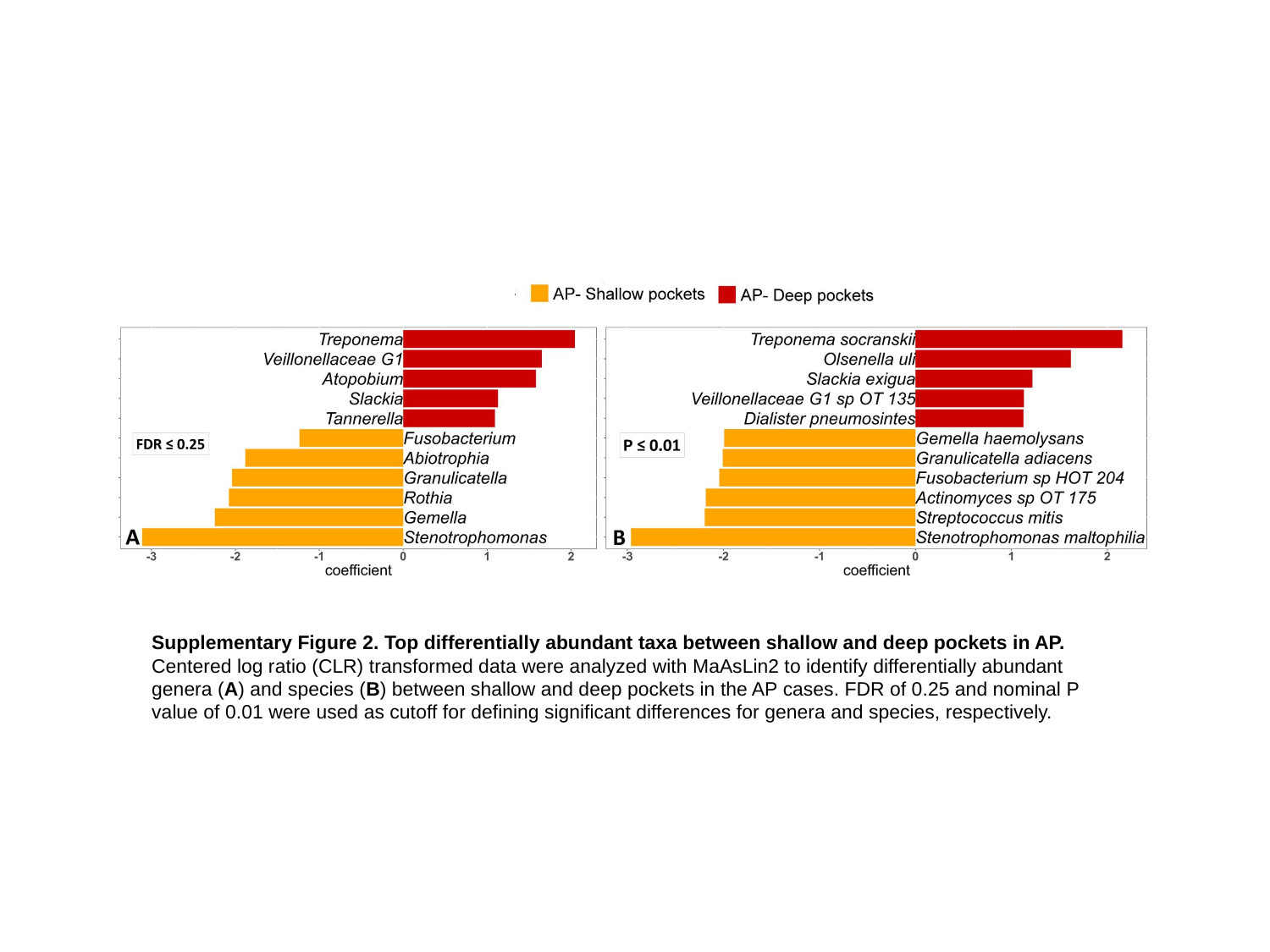

Supplementary Figure 2. Top differentially abundant taxa between shallow and deep pockets in AP. Centered log ratio (CLR) transformed data were analyzed with MaAsLin2 to identify differentially abundant genera (A) and species (B) between shallow and deep pockets in the AP cases. FDR of 0.25 and nominal P value of 0.01 were used as cutoff for defining significant differences for genera and species, respectively.
